# Folie à deux as a cusp catastrophe in coupled active inference: a dynamical systems account of shared psychotic disorder

**DOI:** 10.3389/fpsyt.2026.1886942

**Published:** 2026-08-10

**Authors:** Dustin Beasley

**Affiliations:** School of Psychology, Golden Gate University, San Francisco, CA, United States

**Keywords:** active inference, computational psychiatry, cusp catastrophe, dyadic psychopathology, dynamical systems, *folie à deux*, network physiology, precision weighting

## Abstract

Prior work in computational psychiatry has identified three failure modes of precision weighting that produce the phenomenology of individual-level psychosis: aberrant precision on priors, aberrant precision on sensory likelihood, and aberrant meta-precision. The coupled, dyadic case has remained outside this framework. We identify a fourth failure mode, dyadic precision migration, in which precision migrates from individual sensory and prior channels onto the coupling channel under conditions of external precision collapse and sustained allostatic load. Mode 4 is a supervenient property of the coupled information geometry and is undefined in the decoupled limit; it is invisible to single-agent models of psychosis. Folie à deux, or shared psychotic disorder, is the canonical clinical instance. Starting from coupled active inference, we derive the reduced order-parameter dynamics via Landau theory expansion and show that asymmetric coupling unfolds the symmetric pitchfork bifurcation into the cusp catastrophe normal form. The bifurcation parameter maps to coupling stiffness; the symmetry-breaking parameter maps to coupling asymmetry. Numerical simulations confirm the characteristic semicubical parabola bifurcation geometry and establish that the shared attractor regime is robust (17.8% to 23.4% of explored parameter space) across coupling functional forms and noise distributions. Hysteresis on the cusp manifold predicts a falsifiable ordering of recovery times by the depth to which the secondary’s beliefs are reconfigured: shallow (inference-dominated) cases recover quickly on separation, deep (learning-dominated) cases recover slowly, and cases reflecting two independent pathologies do not remit on separation alone. The framework yields a transdiagnostic account of dyadic delusional convergence and clarifies why folie à deux is structurally rare without requiring rare etiological ingredients.

## 1 Introduction

Two individuals in close proximity come to share a delusional belief. The secondary holds the belief with no independent evidence; removing the secondary from the primary frequently, though not always, resolves the delusion. First named by Lasègue and Falret (1) as *folie à deux*, the phenomenon has been continuously documented for a century and a half (2, 3), codified in diagnostic manuals as shared psychotic disorder (DSM-5 code 298.8) or induced delusional disorder (ICD-11), and refined into four classical subtypes: folie imposée, folie communiquée, folie simultanée, and folie induite. The clinical phenomenology is well-characterized. The computational mechanism is not.

This paper provides a formal dynamical systems characterization of the failure. Under the active inference framework (4, 5), belief updating is implemented as precision-weighted minimization of variational free energy across multiple channels (priors, sensory likelihood, coupling to other agents). When coupling becomes the dominant precision channel, either through elevation of the coupling precision or through collapse of external precision under allostatic constraint, the system’s belief dynamics undergo a symmetry-breaking bifurcation into a shared attractor state. Because typical clinical presentations involve asymmetric coupling, the symmetric pitchfork bifurcation unfolds into the cusp catastrophe, Arnold’s *A*_3_ classification (6). Different coupling configurations correspond to distinct regions of the cusp manifold and yield testable predictions about recovery dynamics.

The contribution is fourfold. First, we derive the cusp catastrophe as the principled reduced normal form of coupled active inference via Landau theory order-parameter expansion; the parameter mapping is explicit. Second, we identify a fourth dyadic precision-weighting failure mode, *precision migration*, that is a supervenient property of the coupled information geometry, not reducible to individual-level precision failures. Third, rather than relying on the historical fourfold subtype taxonomy (which current nosologies do not adopt), we anchor a falsifiable prediction on the depth to which the secondary’s beliefs are reconfigured, ordering recovery times as *T*_shallow_ *< T*_deep_ ≪ *T*_independent_, with the independent pathology case not remitting on separation. Fourth, we verify the dynamical predictions through simulation.

The clinical and psychoanalytic operationalization, including the four-layer decomposition of dyadic coupling and integration with Britton’s (7, 8) theory of triangular space, is developed in a companion paper (Beasley, in preparation).

## 2 The argument in clinical terms

This section states the paper’s claim and its key constructs in plain clinical language before the formal development. Readers fluent in the computational vocabulary may proceed directly to the next section. A glossary mapping each formal term to its plain clinical meaning is given in **Table 1**.

**Table 1:** Mapping between the paper’s formal constructs and their clinical meaning.

Formal term	Plain clinical meaning
Precision (weighting)	How much confidence or reliability the brain assigns to a source of information
Belief difference δ (order parameter)	How far apart the two people’s beliefs are; δ = 0 means identical beliefs
Coupling strength K	How strongly the two people’s beliefs influence each other
Coupling asymmetry K12 − K21	Whether influence is one-sided (primary → secondary) or mutual
External precision πext	Weight available for outside (non-partner) information; collapses under isolation
Bifurcation/tipping point	The threshold past which shared belief abruptly becomes the stable state
Cusp catastrophe	The specific tipping point shape that yields rarity, abrupt onset, and persistence
Hysteresis	Why the shared delusion persists after the original trigger is gone
Bistable region	The range in which both “separate beliefs” and “shared delusion” are stable
Mode 4 (dyadic precision migration)	The two-person failure: confidence migrates onto the partner and off the world
Reconfiguration depth	Whether the partner merely shifted attention (shallow) or durably changed beliefs (deep)

*The claim*: Folie à deux arises when two people, under specific conditions, largely stop weighing the outside world and weigh each other instead, so their beliefs collapse onto a single shared position. We show that this is the expected behavior of two coupled belief-updating systems near a tipping point and that the tipping point has a particular mathematical shape (a *cusp*) from which three clinically familiar features follow automatically: the condition is rare; it tends to appear relatively abruptly; and once established, it persists even after the original trigger is removed.

*The conditions*: These are the recognized risk factors, restated as mechanisms: social isolation (little outside information to compete with the partner), a primary with an independent psychotic illness (a fixed, confident source to lock onto), and a suggestible or dependent secondary (someone for whom deferring to the partner is the path of least resistance).

*What is new*: Existing computational accounts of psychosis describe one brain at a time. This paper adds a failure mode that exists only *between* two coupled people and is invisible when either is examined alone. It also predicts how quickly a secondary should recover after separation: quickly if the partner merely shifted what the secondary attended to (a change of inference), slowly if the partner durably rewired the secondary’s beliefs over time (learning), and not at all on separation alone when the two had genuinely independent illnesses that happened to synchronize.

## 3 Background

### 3.1 The active inference framework

Under the active inference framework (4, 5), an agent models the world through a generative model and updates beliefs by minimizing variational free energy *F*, an upper bound on surprisal. Belief updating proceeds as (Equation 1):

(1) $$\frac{\text{d}q}{\text{d}t}=-\pi_{\text{prior }}\nabla F_{\text{prior}}-\pi_{\text{sensory }}\nabla F_{\text{sensory}}-\cdots$$

where *q* is the posterior belief distribution and the precision weights *π* encode estimated channel reliability. Precision weighting is implemented neurobiologically through neuromodulatory gain control (9, 10).

### 3.2 The three individual-level failure modes

Adams et al. (9) proposed that psychotic symptoms can be understood as specific failures of precision weighting:

Mode 1: Aberrant precision on priors. Pathologically elevated prior precision causes predictions to dominate incoming sensory evidence, producing the phenomenology of delusion.

Mode 2: Aberrant precision on sensory likelihood. Pathologically elevated or noisy sensory precision causes the agent to treat noise or hallucinatory content as veridical, producing hallucination.

Mode 3: Aberrant meta-precision. Failure of contextual precision modulation (10) produces attentional dysregulation and failures to update.

All three modes are defined and measurable at the level of the single agent.

### 3.3 The coupled case as an open problem

The framework of Adams et al. (9) does not address the dyadic case. Friston and Frith (11) modeled healthy coupled active inference and showed that shared beliefs emerge through generalized synchrony. Friston et al. (12) extended this to federated inference. Neither addresses pathology. Folie à deux poses this gap precisely: a pathological configuration that emerges from coupling, not from intrinsic individual precision failure.

## 4 The coupled free energy functional and the fourth failure mode

In plain terms: we write down how each person’s beliefs update under three influences—their own senses, their own prior expectations, and their partner—and then show what happens when the partner’s influence comes to dominate the other two.

### 4.1 Coupled active inference

For a dyadic system with agents *i* ∈ {1,2}, the total variational free energy decomposes as:

(2) $$F_{\text{total}}=F_{1}[q_{1};\;s_{1}]+F_{2}[q_{2};\;s_{2}]+F_{\text{coupling}}[q_{1},q_{2};\;s_{\text{shared}}]$$

where *q_i_* is agent *i*’s posterior belief, *s_i_* their private sensory input, and *s*_shared_ represents shared environmental input. Each agent’s belief updating follows (Equation 3):

(3) $$\frac{\text{d}q_{i}}{\text{d}t}=-\pi_{\text{prior},i}\;\nabla F_{\text{prior},i}-\pi_{\text{sensory},i}\;\nabla F_{\text{sensory},i}-\pi_{\text{coupling},ij}\;\nabla F_{\text{coupling}}$$

### 4.2 Precision migration under allostatic constraint

Under high allostatic load and external precision collapse, the minimum free energy solution is (Equation 4):

(4) $$\pi_{\text{coupling},ij}\to \text{dominant},\;\pi_{\text{sensory},\;}\pi_{\text{prior}}\to \text{relatively downweighted}$$

The system treats the partner’s outputs as the highest-reliability available signal, driving *q*_1_ → *q*_2_. This is the rational Bayesian update under degenerate input conditions, not a malfunction.

The three clinically recognized risk factors for folie à deux (2, 3) map directly onto this configuration, so the migration is grounded in the epidemiology rather than postulated. *Social isolation* is the collapse of external precision (*π*_ext_ ↓): it removes the external sources that would otherwise compete with the coupling channel, lowering the stiffness *a*(*K,π*_ext_) toward the symmetry-breaking threshold. A primary with an independent psychotic disorder is an agent already resident in a biased attractor (elevated prior precision; mode 1 of Adams et al. (9), supplying the dominant high-precision signal on the coupling channel. A suggestible or dependent secondary is precisely the precision configuration (relatively low prior precision, elevated coupling precision) under which migration onto the coupling channel is the free-energy-minimizing update. “High allostatic load” is operationalized as proximity to the bifurcation threshold, for which the standard early-warning signature of critical slowing down (13) is the measurable correlate.

### 4.3 The fourth failure mode as a supervenient property

Mode 4: Dyadic precision migration. Under external precision collapse and high allostatic load, precision migrates from individual channels onto the coupling channel, producing convergence onto a shared attractor state.

Migration operates on two timescales that must be distinguished. The *fast* component is *inference*: within episode precision reweighting onto the coupling channel, in which the partner’s outputs are transiently treated as the highest-reliability signal. It is reversible—removing the coupling reverses it. The *slow* component is *learning*: under sustained coupling, the secondary’s priors (and the likelihood mapping) are durably reconfigured, and this persists after the coupling is removed. This inference/learning distinction is not incidental; it supplies the mechanism for the recovery time predictions below (shallow, inference-dominated couplings relax quickly on separation; deeply learned reconfigurations do not).

Unlike modes 1 to 3, mode 4 is undefined in the decoupled limit (*K* → 0); it is a supervenient property of the dyadic information geometry. A physical analogy: Temperature supervenes on molecular kinetic energy, but no individual molecule has a temperature. Mode 4 cannot be characterized by examining either agent in isolation, which is why folie à deux is invisible to single-agent models of psychosis.

## 5 Reduction to the cusp catastrophe normal form

In plain terms, near the tipping point, the two-person system simplifies to a single, well-studied equation (the cusp), and we identify exactly which clinical quantities act as the two “knobs” that drive it—the strength of mutual influence and its one-sidedness.

### 5.1 Change of coordinates

Define symmetric and antisymmetric modes of the coupled belief dynamics:

(5) $$\sigma =\frac{q_{1}+q_{2}}{2},\;\delta =\frac{q_{1}-q_{2}}{2}$$

The symmetric mode *σ* evolves on a slow manifold near the transition; the antisymmetric mode *δ* carries the symmetry-breaking information. The question “do the agents hold distinct beliefs or a shared belief?” reduces to whether *δ* = 0.

### 5.2 Order-parameter identification

Near the symmetry-breaking transition, *δ* becomes the Landau order parameter: its relaxation time diverges, its dynamics dominate near the transition, and the effective free energy expands as a power series in *δ*.

### 5.3 Expansion of the effective free energy

Expanding $F_{\text{eff}(\delta )}$ around *δ* = 0:

(6) $$F_{\text{eff}}(\delta )=a(K,\pi_{\text{ext}})\;\delta^{2}+b\;\delta^{4}+c(K_{12}-K_{21})\;\delta +O(\delta^{6})$$

where $a(K,\pi_{\text{ext})}$ is the stiffness coefficient (its sign determines stability of *δ* = 0), *b >* 0 is the quartic stabilizing coefficient, and *c*(*K*_12_ − *K*_21_) is the symmetry-breaking coefficient proportional to coupling asymmetry. The *δ* → −*δ* symmetry that would eliminate all odd-order terms holds only in the exactly exchangeable case *K*_12_ = *K*_21_. Asymmetric coupling breaks that symmetry, and the linear term *c*(*K*_12_ − *K*_21_)*δ* is precisely the symmetry-breaking term—it is the formal content of the statement that asymmetric coupling unfolds the pitchfork into the cusp. The cubic term is removed by the standard shift of origin that brings the quartic to canonical cusp form; this is why the linear term is retained while the cubic is not.

### 5.4 Reduction to cusp normal form

The Euler–Lagrange equation for the order-parameter dynamics is:

(7) $$\dot{\delta }\propto -\frac{\partial F_{eff}}{\partial \delta }=-2a\;\delta -4b\;\delta^{3}-c$$

Rescaling $x=\delta \sqrt{2b}$ and $t^{\prime }=2b\;t$:

(8) $$\dot{x}=r\;x-x^{3}-h$$

with the explicit parameter mapping (Equations 9 and 10):

(9) $$r=-\frac{a(K,\pi_{\text{ext})}}{\text{const}}\;(\text{bifurcation parameter})$$

(10) $$h\propto c(K_{12}-K_{21})\;(\text{symmetry}-\text{breaking parameter})$$

Equation 8 is the cusp catastrophe normal form (6, 14, 15). We emphasize that the cusp is not specific to the free-energy functional: it is the generic normal form for any scalar order parameter undergoing symmetry breaking with an asymmetry term, and an analogous reduction holds for a broad class of coupled objective functions parameterized by the belief difference *δ*. The free-energy functional does not *produce* the cusp; what active inference contributes is the *interpretation* of the control parameters—the bifurcation parameter *r* as coupling stiffness *a*(*K,π*_ext_) and the asymmetry parameter *h* as coupling asymmetry *c*(*K*_12_ − *K*_21_)—together with the identification of the fourth dyadic precision-weighting failure mode. The contribution is this mapping, not a claim of free-energy uniqueness.

### 5.5 The symmetric limit recovers the pitchfork

When *K*_12_ = *K*_21_, so *h* = 0 (Equation 11): :

(11) $$\dot{x}=r\;x-x^{3}$$

with symmetric attractors $x^{*}=\pm \sqrt{r}$ for r>0. This symmetric limit is an idealization (exactly exchangeable agents). We caution that it is *not* the clinical *simultanée* presentation, which involves two largely independent psychotic processes rather than symmetric symmetry-breaking from a shared baseline; that case is treated separately below.

## 6 Numerical verification

### 6.1 Cusp geometry verification

For the cusp normal form $\dot{x}=rx-x^{3}+h$, the bistable region is bounded analytically by the semicubical parabola (Equation 12):

(12) $$h^{2}=\frac{4}{27}\;r^{3},\;r\geq 0$$

with cusp point at the origin (*r*, *h*) = (0, 0).

We performed a 250 × 250 grid sweep over (*r,h*) ∈ [−1.2, 2.8] × [−2.2, 2.2]. At each point, we solved the equilibrium condition *x*^3^ − *rx* − *h* = 0 analytically, retained real roots, and counted stable equilibria (those satisfying $f^{\prime }(x)=r-3x^{2}<0$). The analytical bifurcation set (12) was overlaid for comparison.

**Figure 1:**
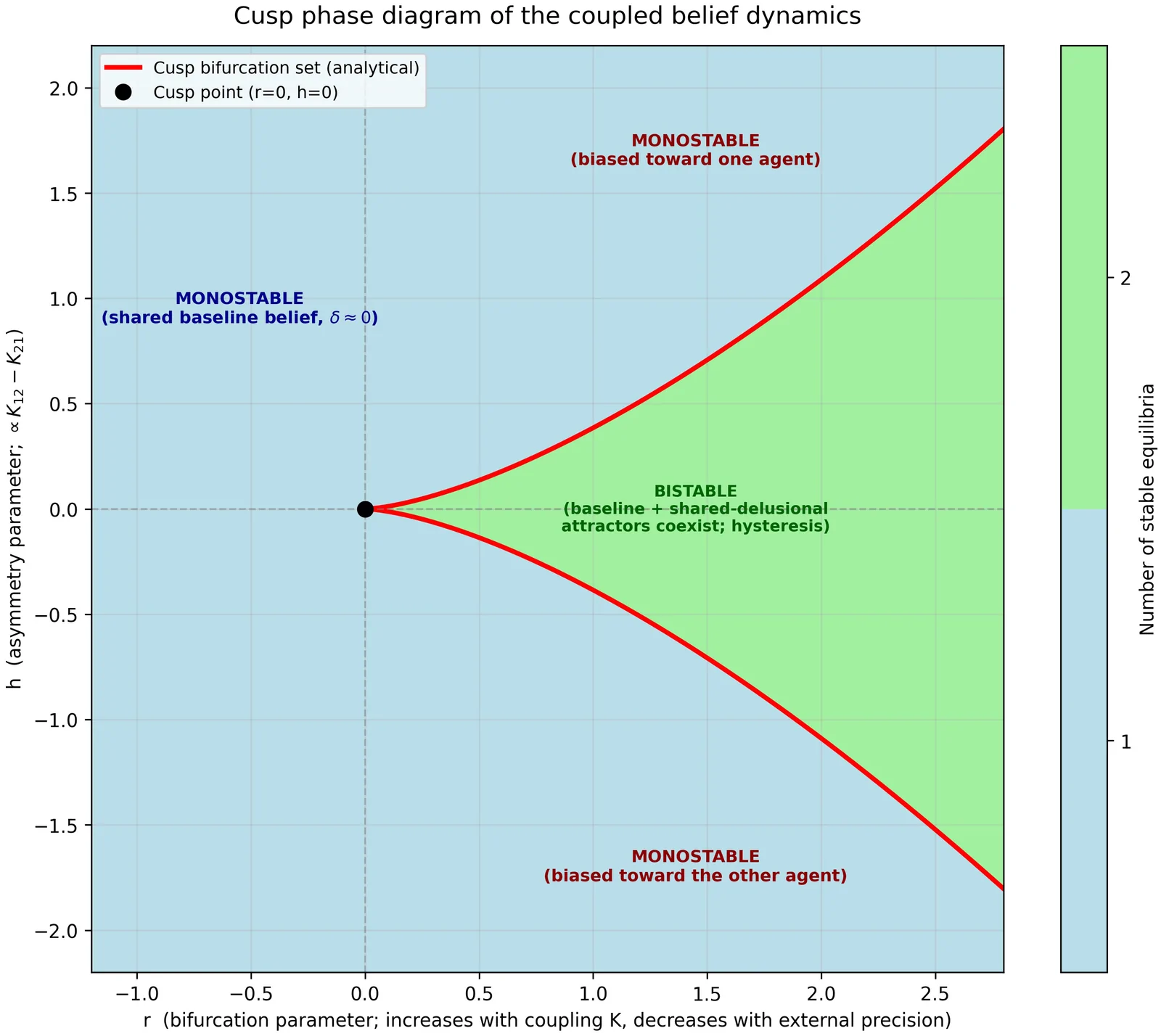
Cusp phase diagram of the coupled belief dynamics in (*r,h*) parameter space, derived from coupled active inference via Landau order-parameter expansion. The bifurcation parameter *r* increases with coupling strength *K* and decreases with external precision; the asymmetry parameter *h* ∝ *K*_12_ − *K*_21_. The cusp bifurcation set (red) follows the semicubical parabola *h*^2^ = (4*/*27)*r*^3^, confirming cusp geometry, with the cusp point at the origin. The bistable region (green) is where a shared delusional attractor coexists with the baseline, permitting abrupt onset and hysteresis; the monostable regions (blue) are the shared baseline belief (*δ* ≈ 0, left) and two biased single-attractor states (upper and lower) in which the shared state is pulled toward one agent. The sign of *h* selects which agent; clinically, this is the primary (the agent with the independent psychotic process). Here, *δ* denotes the belief difference between the agents; the region labels are model definitions, while the empirical predictions are the transition features (bimodality, hysteresis, critical slowing down).

**Figure 2:**
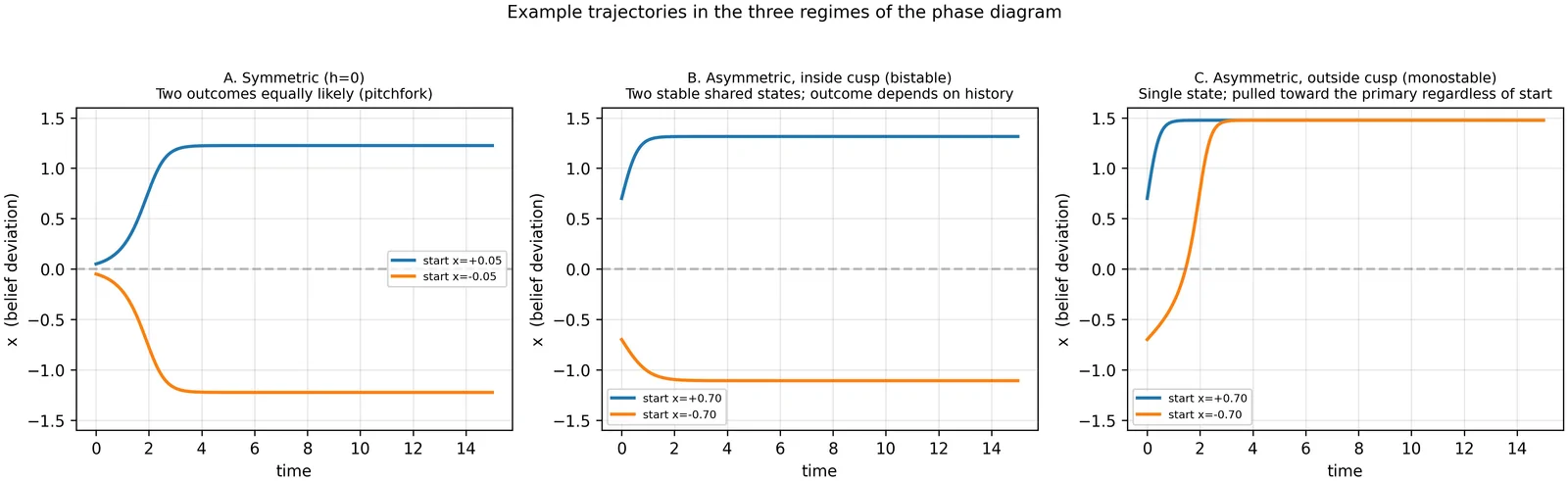
Example trajectories (no noise), one per regime of the phase diagram. **(A)** Symmetric case (*r* = 1.5, *h* = 0): the two initial conditions split to opposite stable branches—two outcomes equally available (pitchfork). **(B)** Asymmetric, inside the cusp (*r* = 1.5, *h* = 0.3): the two initial conditions settle on *two different* stable shared states, so the outcome is history-dependent (bistability). **(C)** Asymmetric, outside the cusp (*r* = 1.5, *h* = 1.0): both initial conditions converge to the *same* single attractor, pulled toward the primary regardless of starting point (monostability). In **(A)**, the initial conditions are *x*_0_ = ± 0.05; in **(B, C)**, *x*_0_ = ± 0.7.

The simulated bistable region is bounded exactly by the semicubical parabola, confirming *r*^3^*^/^*^2^ scaling and the codimension-2 cusp point at the origin (**Figure 1**). Deterministic trajectories integrated from *x*_0_ = ± 0.05 confirm path dependence and hysteresis (**Figure 2**).

### 6.2 Robustness of the narrow closed-loop regime

We performed Euler–Maruyama integration of (Equation 13):

(13) $$\text{d}x=[r(K,E)\;x-x^{3}]\text{ d}t+\text{d}W_{t}$$

on a 40 × 40 grid in (*K,E*) ∈ [0,1]^2^ under four model variants:

- Linear coupling: *r* = *K* − 1.55*E*
- Sigmoidal coupling: $K_{\text{eff}}=K/(1+e^{-8(K-0.35)})$, then *r* = *K*_eff_ − 1.70*E*

**Figure 3:**
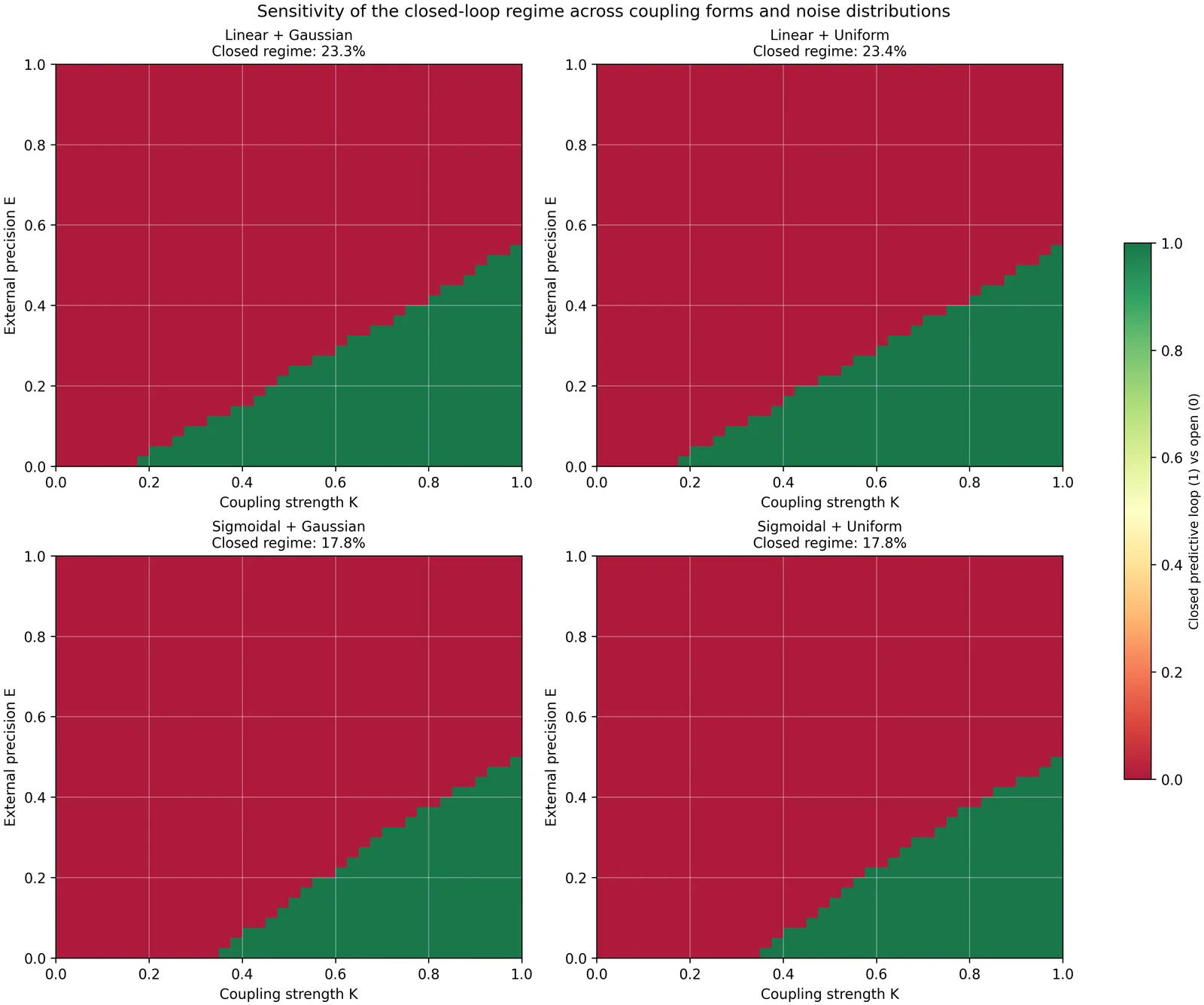
Sensitivity analysis: narrow closed-loop regime across coupling forms (linear vs. sigmoidal) and noise distributions (Gaussian vs. uniform). Green: closed predictive loop; red: open. The Goldilocks zone (17.8% to 23.4%) persists across all four variants, confirming robustness of the rarity prediction.

Gaussian noise: $\text{d}W_{t}\sim N(0,\;1)\text{d}t$Uniform noise: $\text{d}W_{t}\sim U(-1,\;1)\text{d}t$ The closed-loop state was defined as $|x_{\text{final}}|>0.40$ after *T* = 55 time units. Results across all four variants are shown in **Table 2** and **Figure 3**.

**Table 2:** Closed-loop regime across coupling and noise variants.

Coupling	Noise	Closed-loop regime
Linear	Gaussian	23.3%
Linear	Uniform	23.4%
Sigmoidal	Gaussian	17.8%
Sigmoidal	Uniform	17.8%

The narrow Goldilocks zone (17.8% to 23.4%) persists across all variants, with <6 percentage points of variation. This structural narrowness provides a mechanistic account of the clinical rarity of folie à deux that is not an artifact of single-variant parameter choices.

## 7 From historical subtypes to reconfiguration depth

### 7.1 Why not the fourfold taxonomy

The historical French subtypes (imposée, communiquée, simultanée, induite) are descriptive labels. Neither DSM-5-TR nor ICD-11 adopts the fourfold scheme, and it has not been epidemiologically validated (2, 3). We therefore do not treat the subtypes as a nosology or rest predictions on them. Instead, we anchor the model’s prediction on a continuous, clinically grounded variable—the *depth to which the secondary’s generative model has been reconfigured* by the coupling—which maps directly onto the recognized risk factors (a primary with an independent psychotic disorder, a suggestible or dependent secondary, and social isolation).

### 7.2 Three structural configurations

Shallow/inference-dominated (the imposée-type configuration). Precision is transiently reweighted onto the coupling channel; the secondary’s priors are not durably altered. This is an inference change, reversible on decoupling. Prediction: separation removes the coupling and the secondary’s beliefs relax quickly; shortest recovery.

Deep/learning-dominated (the communiquée-type configuration). Sustained coupling has durably reconfigured the secondary’s prior structure: this is learning, not merely inference, and it persists after the coupling is removed. Prediction: separation is necessary but not sufficient; recovery is slow and additional intervention is typically required.

Independent-pathology (the clinical simultanée configuration). Two individuals each already resident in an independent-biased attractor (each with their own psychotic process) phase-lock—generalized synchrony of coordinate-independent pathologies rather than symmetry-breaking from a shared baseline. Prediction: under separation (*K* → 0), each agent remains in its pre-existing attractor; neither remits spontaneously, and separation serves to enable independent (e.g., pharmacological) treatment rather than to resolve the delusion in itself. This accords with the clinical observation that simultaneous cases do not remit on separation alone.

The remaining historical category, *folie induite* (emergence of novel belief content in the secondary), involves new attractor states that exceed the present two-variable reduction and is left to a hierarchical extension.

### 7.3 The recovery-depth prediction

The primary falsifiable prediction is an ordering by reconfiguration depth rather than by historical label:

(14) $$T_{\text{shallow}}<T_{\text{deep}}\ll T_{\text{independent}},$$

where the independent pathology case does not remit on separation at all. The ordering follows from basin depth on the manifold and from the inference-versus-learning distinction introduced above. It is testable via retrospective case series scored on the recognized risk factors and on indices of how durably the secondary’s beliefs were reconfigured (e.g., duration of coupling, persistence of belief after separation, presence of independent psychotic illness in each party).

## 8 Related work

### 8.1 Cusp catastrophe in individual psychiatry

Lange and Houran (16) operationalized Maher’s attributional theory of delusions as a cusp catastrophe. Giotakos (17) extended cusp analysis to psychosis broadly. Guastello (18) surveyed catastrophe theory across clinical psychology. All three remain at the individual level.

### 8.2 Coupled-dyad dynamical modeling

Nowak and Vallacher (19) pioneered coupled-dyad dynamical modeling of close relationships. Kelso et al. (20)Kelso (21) developed the HKB framework for coupled human systems, extended to cognitive and social coordination (22, 23). These provide methodological precedent but do not address pathology.

### 8.3 Catastrophe theory in clinical dyadic pathology

Katerndahl et al. (24, 25 established cusp catastrophe for dyadic clinical pathology in intimate partner violence. Gottman et al. (26) applied catastrophe theory to marital dissolution. Our framework shares the mathematical machinery but targets dyadic *convergence* (shared belief formation) rather than divergence (relationship dissolution); these are topologically inverse phenomena on the same cusp landscape.

### 8.4 Active inference in psychosis and shared cognition

Adams et al. (9) provided the computational anatomy of individual psychosis. Friston and Frith (11) modeled healthy coupled active inference. Friston et al. (27) labeled a convergent regime of their active-inference dialogue simulator as “folie à deux” (Figure 13 caption; Section 5.2), describing it as an “unstable fixed point of uncertainty that converges onto a shared fantasy,” without analyzing its bifurcation structure. The present framework provides the formal dynamical characterization that their descriptive label invokes.

### 8.5 Circular inference

Circular inference (28) models *vertical* hierarchical reverberation within a single brain. Our closed predictive loop models *horizontal* reverberation between two coupled agents. The two mechanisms are complementary.

### 8.6 Technological folie à deux

Dohnány et al. (29) introduced the concept qualitatively. Lipinska and Brosnahan (30) proposed an ontological dissonance hypothesis. Chandra et al. (31) provided formal Bayesian modeling demonstrating vulnerability to delusional spiraling even in idealized Bayesian users. Our continuous dynamical model characterizes the structural properties of the absorbing attractor underlying the threshold-crossing behavior of Chandra et al.

## 9 Discussion

### 9.1 Why active inference specifically

Active inference is a natural framework for this analysis, but we do not claim that it is uniquely necessary. The cusp reduction holds for a broad class of coupled agents that minimize a belief-difference-parameterized objective with reliability (precision) weighting; this class includes bounded-rational Bayesian reinforcement learning and, for two agents, interactive POMDP formulations, in which an energetic/allostatic cost is likewise central to the analysis. We adopt active inference because it is the most parsimonious instantiation *for psychosis specifically*: precision weighting is the established computational vocabulary for delusion and hallucination (9), giving mode 4 a direct lineage to the individual-level account. We make no claim that features unique to active inference, as against Bayesian RL or I-POMDP, are required for the result; the contribution is the dyadic precision migration mode and its cusp reduction, which any of these instantiations can carry.

### 9.2 Absorbing precision configuration

Once the dyad enters the bistable interior of the cusp manifold, the relative precision on external channels is low *by construction*: it is the same precision configuration—coupling-channel dominance under collapsed *π*_ext_— that produced the transition, not a separate assumption. External signals continue to arrive but are weighted ≈0 in the posterior update. We term this regime the *absorbing precision configuration*: the system cannot incorporate exogenous information without first undergoing a structural change in precision allocation, that is, without first leaving the cusp region. We are careful not to claim that this *explains* the imperviousness of delusion to argument: delusion is definitionally resistant to disconfirmation, so such a claim would be circular. We claim only a *structural* account of why, in the coupled case, disconfirming input is down-weighted—it is a property of the precision configuration rather than of the propositional content—which is consistent with the clinical ineffectiveness of content-focused argumentation.

### 9.3 Extension to other coupled-agent pathologies

The framework is not specific to folie à deux. Any coupled-agent configuration in which one channel dominates precision allocation under external collapse and allostatic constraint should exhibit the same cusp structure. Candidate extensions include cult-induced belief states, intensive coaching dyads, and human–AI interactions in which an always-available artificial interlocutor supplies the dominant precision source under conditions of user isolation (29, 31). We note these as testable extensions rather than developing them here.

### 9.4 Relation to network physiology

The present framework is a social- and cognitive-scale instance of the organizing principle at the center of network physiology (32, 33): integrated states arise from the dynamic coupling and synchronization of distinct units, and transitions between states are marked by a reorganization of that coupling. Network physiology characterizes how physiologic systems (cardiac, respiratory, neural, locomotor) dynamically interact, synchronize, and exchange information across scales, with health and pathology distinguished by network topology and its reconfiguration (32, 34). Our account applies the same lens one level up, to two coupled inferential agents: belief synchronization is the order parameter, precision-weighted coupling is the information flow channel, the inference-versus-learning separation is a multiscale (fast/slow) decomposition, and dyadic convergence onto a shared attractor is a collective dynamics transition. The organizing themes of this Research Topic—synchrony, information flow, multiscale coupling, and collective dynamics—thus map directly onto the paper’s constructs, with folie à deux appearing as the pathological limit of interagent synchronization. This positions dyadic psychopathology within the broader network physiology program (33) and makes the framework’s empirical predictions—physiological synchronization measured by HRV and EEG hyperscanning, and critical slowing down approaching lock-in—directly addressable with the measurement toolkit of network physiology.

### 9.5 Clinical implications

The full clinical operationalization is developed in a companion paper. We stress that the core structural principle—that effective intervention is structural rather than content-focused, with separation and cognitive distance between the parties as first-line treatment—is long established in the clinical literature and is *not* a novel implication of this analysis. What the present model adds is twofold: first, a candidate *monitoring* target, the critical slowing-down early-warning signature (rising variance and autocorrelation of belief-state measures) as the dyad approaches lock-in (13); and second, a guide to *expected recovery trajectory and intervention intensity* via the reconfiguration-depth distinction (shallow vs. deep vs. independent pathology). We claim novelty only for these, not for the separation principle itself.

## 10 Falsifiability and limitations

### 10.1 Primary falsification criterion

Equation 14 is the primary falsifiable prediction. It is falsified if cases with shallow, inference-dominated coupling (brief coupling duration, no independent psychotic illness in the secondary) consistently show *slower* recovery on separation than cases with deep, learning-dominated reconfiguration; or if cases reflecting two independent pathologies are observed to remit spontaneously on separation alone.

### 10.2 Secondary falsification criterion

In longitudinal dyad studies, variance and autocorrelation of belief-state measures should rise sharply near lock-in, following the early-warning signature of Scheffer et al. (13) for bifurcation transitions.

### 10.3 Limitations

(1) The two-variable reduction is minimal; hierarchical extension is natural follow-up. (2) The framework is theoretical; empirical validation (HRV hyperscanning, EEG coherence, longitudinal belief tracking) is scheduled follow-up work. (3) The parameter regime estimate (17.8% to 23.4%) depends on chosen coupling forms and noise distributions. (4) Folie à deux has low base rate (≈0.006% general population; ≈1.5% inpatient); prospective studies sufficient to detect early-warning signatures may be statistically challenging.

### 10.4 Falsifiability under the free energy principle critique

The FEP has been criticized as unfalsifiable in its most general formulations (35, 36). We emphasize that the present paper proposes a *process theory*, which is a specific instantiation with explicit parameters and falsifiable predictions, rather than the FEP in its general form.

## 11 Conclusion

We have provided a formal dynamical systems account of folie à deux as a cusp catastrophe in coupled active inference with asymmetric precision weighting. The cusp emerges as the reduced normal form via Landau theory order-parameter expansion; the parameter mapping is explicit and verified numerically. The framework identifies a fourth precision-weighting failure mode, dyadic precision migration, which is a supervenient property of the coupled information geometry. Rather than resting on the historical fourfold taxonomy (which current nosologies do not adopt), the model yields a falsifiable prediction ordered by the depth to which the secondary’s beliefs are reconfigured: shallow, inference-dominated cases recover fastest on separation; deep, learning-dominated cases recover slowly; and cases reflecting two independent pathologies do not remit on separation alone.

The mechanism generalizes beyond folie à deux to any coupled-agent configuration that satisfies the precision migration condition. The clinical operationalization, including configuration-specific intervention strategies and integration with psychoanalytic theories of triangular space, is developed in a companion paper.

## Data Availability

The original contributions presented in the study are included in the article/supplementary material. Further inquiries can be directed to the corresponding author.

## Appendix A: Landau theory derivation of the cusp normal form

The derivation in the section “Reduction to the Cusp Catastrophe Normal Form” follows the standard Landau theory reduction as set out in Landau and Lifshitz, *Statistical Physics* §142. Starting from the coupled free energy functional $F_{\text{total}}$ (Equation 2), the antisymmetric mode *δ* (Equation 5) is identified as the order parameter because its relaxation time diverges at the symmetry-breaking transition while the symmetric mode *σ* remains massive. Expanding $F_{\text{eff}}(\delta )$ to fourth order in *δ* with *Z*_2_ symmetry broken by the coupling asymmetry term $c(K_{12}-K_{21})\delta$ yields Equation 6. The gradient-descent dynamics $\dot{\delta }\propto -\partial F_{\text{eff}}/\partial \delta$ yield Equation 7. The rescaling $x=\delta \sqrt{2b},t^{\prime }=2bt$ then gives the cusp normal form Equation 8. The cusp catastrophe is confirmed (rather than a saddle-node) because the system possesses the codimension-2 degeneracy at (r,h)=(0,0) arising from the coupling asymmetry parameter, which is absent in the generic fold/saddle-node family.
